# Characterizing Fluctuations of Arterial and Cerebral Tissue Oxygenation in Preterm Neonates by Means of Data Analysis Techniques for Nonlinear Dynamical Systems

**DOI:** 10.1007/978-1-4939-3023-4_64

**Published:** 2015-06-22

**Authors:** Stefan Kleiser, Marcin Pastewski, Tharindi Hapuarachchi, Cornelia Hagmann, Jean-Claude Fauchère, Ilias Tachtsidis, Martin Wolf, Felix Scholkmann

**Affiliations:** 1 Biomedical Optics Research Laboratory, Department of Neonatology, University Hospital Zurich, University of Zurich, 8091 Zurich, Switzerland; 2 Department of Medical Physics and Bioengineering, University College London, London, UK; 3 Division of Neonatology, University of Zurich, 8091 Zurich, Switzerland

**Keywords:** Long term measurements, Autoregulation, Near infrared spectroscopy, Correlation analysis, Spontaneous fluctuations

## Abstract

The cerebral autoregulatory state as well as fluctuations in arterial (SpO_2_) and cerebral tissue oxygen saturation (StO_2_) are potentially new relevant clinical parameters in preterm neonates. The aim of the present study was to test the investigative capabilities of data analysis techniques for nonlinear dynamical systems, looking at fluctuations and their interdependence. StO_2_, SpO_2_ and the heart rate (HR) were measured on four preterm neonates for several hours. The fractional tissue oxygenation extraction (FTOE) was calculated. To characterize the fluctuations in StO_2_, SpO_2_, FTOE and HR, two methods were employed: (1) phase-space modeling and application of the recurrence quantification analysis (RQA), and (2) maximum entropy spectral analysis (MESA). The correlation between StO_2_ and SpO_2_ as well as FTOE and HR was quantified by (1) nonparametric nonlinear regression based on the alternating conditional expectation (ACE) algorithm, and (2) the maximal information-based nonparametric exploration (MINE) technique. We found that (1) each neonate showed individual characteristics, (2) a ~60 min oscillation was observed in all of the signals, (3) the nonlinear correlation strength between StO_2_ and SpO_2_ as well as FTOE and HR was specific for each neonate and showed a high value for a neonate with a reduced health status, possibly indicating an impaired cerebral autoregulation. In conclusion, our data analysis framework enabled novel insights into the characteristics of hemodynamic and oxygenation changes in preterm infants. To the best of our knowledge, this is the first application of RQA, MESA, ACE and MINE to human StO_2_ data measured with near-infrared spectroscopy (NIRS).

## Introduction

Preterm infants exhibit an immature regulation of respiration as well as systemic and cerebral blood circulation (i.e. cerebral autoregulation, CO_2_ vasoreactivity), leading to an increased incidence of hypoxic and hyperoxic episodes due to (1) large fluctuations in cerebral hemodynamics, and (2) impaired coupling between cerebral blood flow (CBF) and metabolic demand [1]. Episodes of intermittent hypoxemia occur in 74 % of preterm infants, compared to 62 % of term infants [2]. Hyperoxemia or hypoxemia may lead to an increase in mortality and neurological morbidity with long-term effects in later adult life. Greater variability in arterial oxygen saturation (SpO_2_) [3] correlates with an increased incidence of retinopathy of prematurity (ROP). Thus, the assessment of the dynamics of SpO_2_ and cerebral tissue oxygen saturation (StO_2_) in preterm neonates may be of high clinical relevance. Due to continuous advancement in biomedical optics [4, 5], a reliable noninvasive long-term measurement of StO_2_ in preterm neonates is in principle feasible [6, 7].

The aim of the present study was to analyze long-term measurements of StO_2_ (conducted by multi-distance near-infrared spectroscopy, MD-NIRS) and SpO_2_, heart rate (HR) and the fractional tissue extraction (FTOE) in preterm infants by means of data analysis techniques for nonlinear dynamical systems in order to investigate the characteristics of cerebral and systemic hemodynamic fluctuations and their interdependence.

## Material and Methods

### Subjects, Instrumentation and Experimental Protocol

A total of 20 clinically stable preterm neonates were enrolled. The study was approved by the ethics committee, and written informed consent was obtained from the parents before the study. Four neonates were selected for the present analysis, namely those with long continuous signals and the highest signal-to-noise ratio (SNR) (Table 64.1). SpO_2_ and HR were determined by a standard patient monitor (Infinity Delta XL, Dräger, Germany) and StO_2_ by an internally developed MD-NIRS device (OxyPrem, 4 × 3 [760, 805, 870 nm] light sources, two source-detector distances, i.e. 1.5, 2.5 cm [8]). OxyPrem uses the self-calibrating approach [9] which ensures a robust and high-precision measurement of absolute StO_2_ values [10]. The NIRS optode was positioned over the left prefrontal cortex (PFC).

**Table 64.1:** Description of the study sample

Characteristics	Neonate #1	Neonate #2	Neonate #3	Neonate #4
GA at birth (weeks)	33.4	26.4	29.4	26.8
GA at measurement (weeks)	34.7	28.5	29.9	30.7
Weight at measurement (g)	2220	1280	1090	1440
Apgar (1, 5, 10)	8, 8, 9	5, 4, 5	8, 8, 8	5, 8, 8
Respiration	Spontaneous	SIMV	CPAP	Spontaneous
FiO_2_ (%), Hct (%), Hb (g/dL)	21, 50.6, 16.6	25, 40.9, 13.4	21, 49.5, 16.1	21, 36, 11.7
PDA	No	No	Yes	No
Length of analyzed data (min)	111	271	145	308

*GA* gestational age, *FiO*_*2*_ fraction of inspired oxygen, *SIMV* synchronized intermittent mandatory ventilation, *CPAP* continuous positive airway pressure, *Hct* hematocrit, *Hb* hemoglobin, *PDA* persistent ductus arteriosus

Measurements were performed continuously during the night (from ~10 pm till ~6 am), i.e. NIRS measured the resting-state activity of cerebral hemodynamics.

### Signal Processing and Data Analysis

From the SpO_2_ and StO_2_ we calculated the fractional tissue oxygenation extraction (FTOE = (SpO_2_-StO_2_)/SpO_2_) × 100 [%]. FTOE quantifies the balance between oxygen delivery and oxygen consumption and correlates significantly with the invasively measured oxygen extraction fraction [11]. All signals (SpO_2_, StO_2_, FTOE and HR) were downsampled to 0.05 Hz to increase the SNR and since only low frequencies were of interest. For each of the four datasets, an interval was chosen for the subsequent analysis which contains data without any signal distortion. The lengths of the data are given in Table 64.1. To characterize the fluctuations in StO_2_, SpO_2_, FTOE and HR, two different methods were applied:

- *Phase*-*space modeling and application of the recurrence quantification analysis* (*RQA*) [12, 13]. Each signal (StO_2_, SpO_2_, FTOE and HR) was embedded into a phase space with the dimension *m* and time delay *τ*. The optimal values for *m* and *τ* were determined by finding the first minimum of the false nearest neighbors function depending on *m*, and the autocorrelation function depending on *τ*, respectively. In a subsequent step, the phase space trajectories were characterized by the RQA. In particular, the determinism (*DET*, i.e. the predictability of the system), entropy of the diagonal length (*ENT*, i.e. the complexity of the system’s deterministic dynamics), and laminarity (*LAM*, i.e. the amount of intermittency of the system’s dynamics) were calculated.
- *Maximum entropy spectral analysis* (*MESA*) [14]. This method enables a high-precision spectral analysis based on the principle of maximum entropy. To prevent spurious peaks, the order of the MESA-based periodogram was set at one third of the number of samples [15].

The correlation between StO_2_ and SpO_2_ as well as FTOE and HR were quantified by two nonparametric methods:

- *Nonparametric nonlinear regression based on the alternating conditional expectation* (*ACE*) *algorithm* [16]. This technique finds the optimal transformations for the dependent and independent variables in order to maximize the correlation. The correlation strength is quantified by the maximal correlation coefficient, *r*_ACE_.
- *Maximal information*-*based nonparametric exploration* (*MINE*) *technique* [17]. MINE enables the characterization of dependencies between variables. We calculated the maximal information coefficient (*MIC*) (relationship strength) and maximum asymmetry score (*MAS*) (departure from monotonicity).

In addition, each signal was characterized by calculating the median, and variability index 1 (*VI*_1_, quantified as the mean of the modulus of the first derivation). In addition, the relationship of the fluctuation strength of StO_2_ vs. SpO_2_ was determined by the ratio of their standard deviations (variability index 2, *VI*_2_).

## Results, Discussion, Conclusion and Outlook

**Fig. 64.1:**
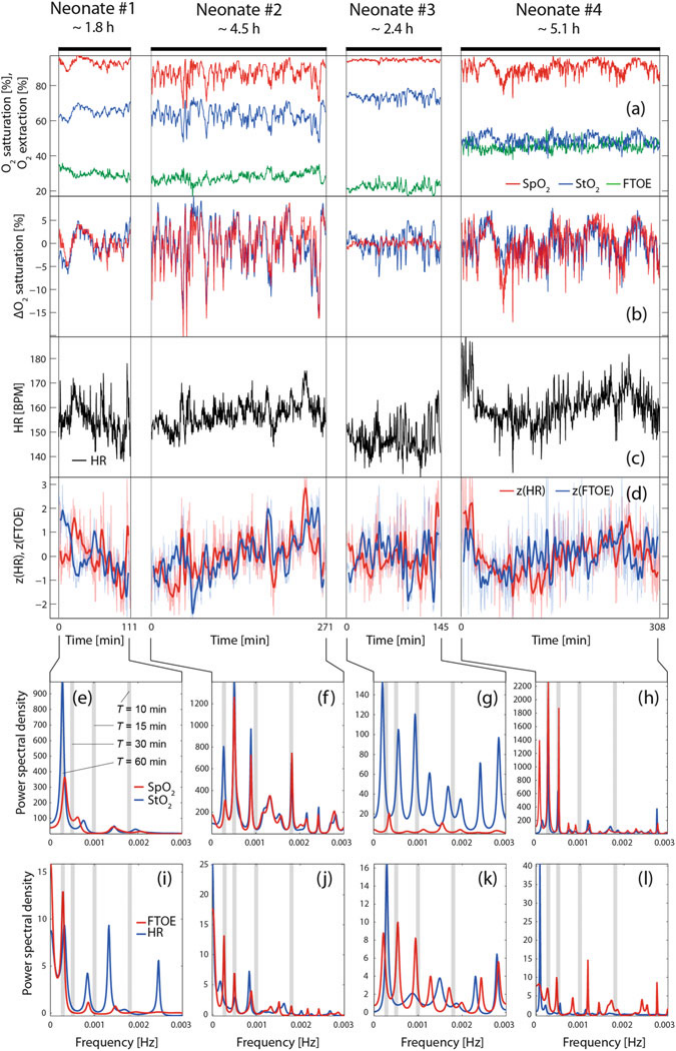
(**a**–**d**) Visualization of the analyzed signals (StO_2_, SpO_2_, FTOE and HR). (**e**–**l**) Frequency spectra obtained by MESA

**Fig. 64.2:**
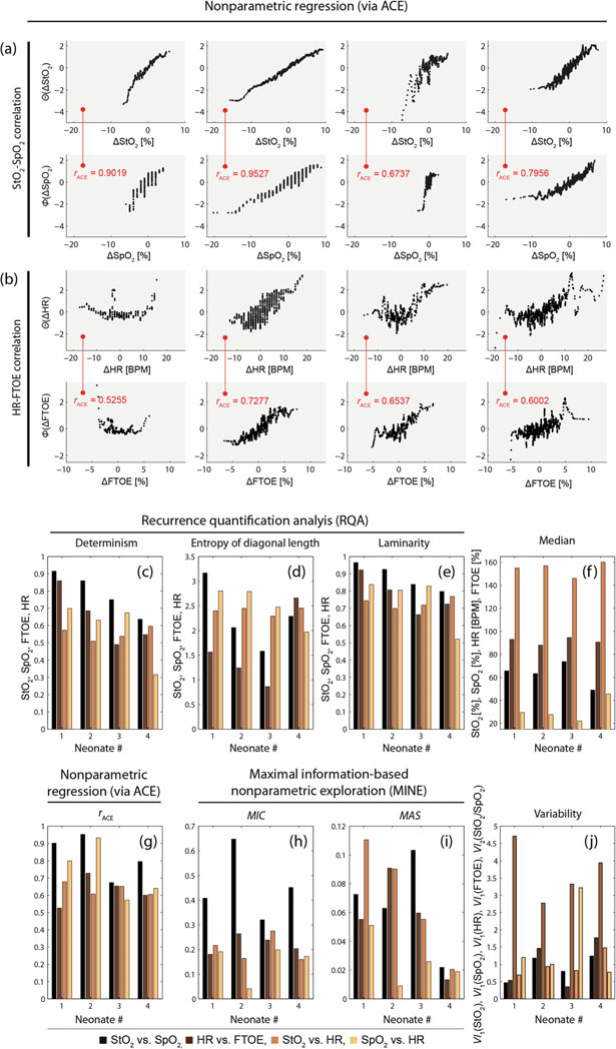
(**a**, **b**) Correlation diagrams based on ACE nonparametric nonlinear regression. (**c**–**j**) Parameters obtained by RQA, MESA, ACE, MINE as well as the values for the median and variability

Figure 64.1a–d shows the time courses of SpO_2_, StO_2_, HR and FTOE. In Fig. 64.1b the normalized (i.e. subtraction of the mean value) SpO_2_ and StO_2_ were plotted to increase the visibility of similar dynamics. Figure 64.1d shows FTOE and HR after normalization (z-score) and smoothing (Kolmogorov-Zurbenko filter, window length: 180 s, iterations: 2) which increases the visibility of the similar long-term variability of both signals. The ACE correlation plots as well as the RQA, ACE and MINE results are visualized in Fig. 64.2. All signals show subject-specific dynamics:

- RQA: Noticeable low values for *DET* and *LAM* in neonate #4, high values for neonate #1. *ENT* (for StO_2_ and SpO_2_) has low values for neonate #3.
- MESA: (1) Neonate #1 exhibited a large oscillation with a period length (*T*) of 60 min in StO_2_ and SpO_2_ as well as HR and FTOE. (2) A large oscillation with *T* ≈ 30 min is present in neonate #2 for StO_2_ and SpO_2_, followed by a second strongest oscillation with *T* ≈ 60 min. In FTOE the predominant oscillation was at *T* ≈ 60 min. The spectra of StO_2_ and SpO_2_ have a remarkably similar fine structure of oscillatory peaks indicating a large degree of similarity in the dynamics. In addition, an oscillation with *T* ≈ 15 min can be seen in StO_2_, SpO_2_, HR and FTOE. (3) Neonate #3 shows an oscillation peak with *T* ≈ 60 min in StO_2_, SpO_2_, HR and FTOE, whereas for FTOE a larger oscillation with *T* ≈ 30 min is present. The spectra of StO_2_ and SpO_2_ have different fine structures, which is also true for the spectra of HR and FTOE. (4) Neonate #4 shows a strong peak with *T* ≈ 60 and 30 min in StO_2_ and SpO_2_.
- ACE and MINE: Concerning the relationship between StO_2_ and SpO_2_, the largest *r*_ACE_ and *MIC* value was found for neonate #2, the lowest for neonate #3; *MAS* was highest in neonate #3. Concerning HR vs. FTOE, neonate #2 had the largest *r*_*ACE*_, *MIC* and *MAS* values. Neonate #4 showed significantly low *MAS* values for all four conditions (StO_2_ vs. SpO_2_ or HR, HR vs. FTOE or SpO_2_).

To interpret the results it is helpful to discuss the similarities and differences of the signal characteristics with respects to the four neonates:

- *Similarities*: (1) The values for *DET*, *ENT* and *LAM* were all higher for StO_2_ compared to SpO_2_ (except for *ENT* of neonate #4), indicating more complex signal characteristics of StO_2_ than of SpO_2_. (2) The correlation between StO_2_ and SpO_2_ is higher than that observed between HR and FTOE (*r*_ACE_(StO_2_, SpO_2_) = 0.8310 ± 0.1236, *MIC*(StO_2_, SpO_2_) = 0.4570 ± 0.1385; *r*_ACE_(HR, FTOE) = 0.6268 ± 0.0854, *MIC*(HR, FTOE) = 0.2216 ± 0.0371). (3) All neonates showed an oscillation in StO_2_, SpO_2_, FTOE and HR with a period of *T* ≈ 60 min, whereas the amplitude was specific for each neonate.
- *Differences*: (1) *DET*, *LAM* and *ENT* of SpO_2_ were highest for neonate #3. (2) The correlation (*r*_ACE_, *MIC*) between StO_2_ and SpO_2_ as well as HR and FTOE was highest for neonate #2; neonate #3 showed the lowest StO_2_/SpO_2_ correlation. (3) The smallest *MAS* values for all four correlations were found for neonate #4 (except for StO_2_ vs. HR, neonate #2). (4) In neonate #3 we observed the highest median values for StO_2_ and SpO_2_ as well as the lowest ones for HR and FTOE. In neonate #4 the lowest StO_2_ and the highest FTOE value were measured. (5) High *VI*_1_ values in StO_2_ and SpO_2_ were present in neonates #2 and #4. Neonate #3 showed the largest *VI*_2_ value for StO_2_/SpO_2_. (6) The mean StO_2_ values correlated inversely with the individual Hct and GA (at birth) (*r* = −0.927 and *r* = −0.982, respectively; *p* < 0.05).

The physiological interpretation of these findings is not straightforward since all patient-specific characteristics have an influence on the analyzed parameters. In particular, the general health state (e.g. PDA, microbleeds, ischemia: yes/no), the type of respiration (ventilatory support: yes/no, type of support), and the GA (at birth/measurement) could potentially have a strong impact on the parameters. The following observations were made based on our analysis: (1) The general inverse correlation observed between StO_2_ and Hct was also noticed by other studies (e.g. [18]). (2) Neonate #3 exhibited a large *VI*_2_, e.g. the fluctuations in StO_2_ were much stronger than in SpO_2_ (especially the decreases), a pattern that is observed by neonates with a PDA—indeed, neonate #3 had a PDA (which was however classified as not hemodynamically relevant). The low StO_2_/SpO_2_ correlation (*r*_ACE_, *MIC*) and the different frequency spectra (StO_2_ vs. SpO_2_, HR vs. FTOE) point also to a specific state of the systemic-cerebral hemodynamic coupling. The observation that neonate #3 had the highest median values for StO_2_ and SpO_2_ as well as the lowest ones for HR and FTOE is surprising since one would expect an increased FTOE and decrease StO_2_ in case of a PDA [19]. (3) The oscillations in the data with *T* ≈ 60 and 30 min could originate from sleep phases. A sleep-wake cycling (with a quiet sleep phase with *T* ≈ 20 min) is known [20] in term newborns with *T* ≈ 50–60 min and an increase in total hemoglobin and HR during active sleep (compared to quite sleep) has previously been observed [21, 22]. (4) The two neonates with the lowest GA at birth (#2, #4) had the largest variability of StO_2_, SpO_2_ and FTOE which could indicate an immature functioning of cerebral hemodynamic regulation.

In conclusion, using four case studies, we demonstrated the possibility of realizing long-term measurements in preterm neonates with MD-NIRS and we presented a novel framework for investigating the characteristics of cerebral and systemic hemodynamic fluctuations and their interdependence. A follow-up study, investigating the signal characteristics in healthy and ill preterm neonates using the same framework would be the next step. Focusing on the fluctuation characteristics of the signals may offer novel insights into systemic and cerebral hemodynamics that are not assessed routinely only using traditional analyses (i.e. based on moments and linear correlations). To the best of our knowledge, this is the first application of RQA, MESA, ACE and MINE to human NIRS data.
